# Near Green Synthesis of Porous Graphene from Graphite Using an Encapsulated Ferrate(VI) Oxidant

**DOI:** 10.1021/acsomega.3c03812

**Published:** 2023-08-03

**Authors:** Bhavya Joshi, Ahmed M.E. Khalil, Tanveer A. Tabish, Fayyaz A. Memon, Hong Chang, Shaowei Zhang

**Affiliations:** † Faculty of Environment, Science and Economy, University of Exeter, Exeter EX4 4QF, U.K.; ‡ Department of Chemical Engineering, Faculty of Engineering, Cairo Universitynal-id id_type=″Ringgold″ id_value=″3286″ source-system=″pplus″/>, Giza 12613, Egypt; § Division of Cardiovascular Medicine, Radcliffe Department of Medicine, University of Oxford, Oxford OX3 7BN, United Kingdom

## Abstract

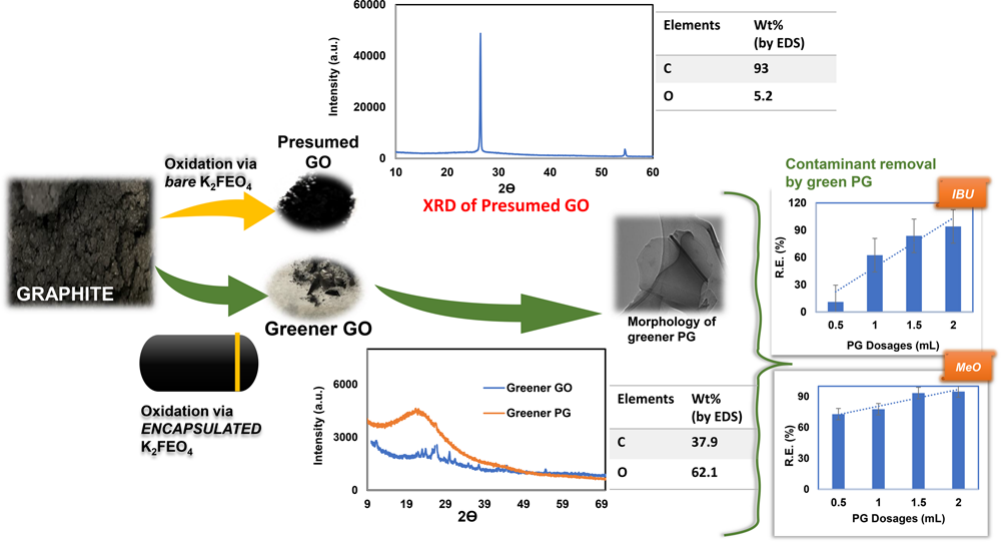

Graphene oxide (GO) is a conventional yet vital precursor for the synthesis of porous graphene (PG). Several strong oxidizing agents such as potassium permanganate and perchlorates are typically used for oxidization of graphite. However, they expose toxic reactants/products that harm the environment. Therefore, a greener approach is desperately needed to oxidize and exfoliate graphite. This study reports for the first time on successful oxidation of graphite by ferrate(VI) compounds via an encapsulation approach. By further reducing GO prepared from this near green route with vitamin C, PG anticipated by many highly important and expanding areas such as water treatment could be readily achieved. X-ray diffraction (XRD), Fourier transform infrared (FTIR) and UV–vis spectroscopy, and scanning electronic microscopy (SEM) along with energy-dispersive spectroscopy confirmed the high yield of GO from the oxidation of graphite. Raman spectroscopy, XRD, and TEM confirmed the formation of high-quality few-layered PG from the reduction of as-prepared GO. The above results demonstrated the practicality of using encapsulated ferrate(VI) compounds to realize green oxidation of graphite and resolve the paradox about the oxidation capability of ferrate(VI). To further illustrate its potential for the removal of emerging and crucial contaminants from water, as-prepared PG was further examined against the contaminants of methyl orange (MeO) dye and ibuprofen (IBU). Taken together, the results revealed that more than 90% removal efficiency could be achieved at a high PG dosage against MeO and IBU. This ground-breaking greener approach opens the door to risk-free, extensive graphene environmental applications.

## 1 Introduction

Water is vital for the sustainability of the ecosystem and human society. Unfortunately, due to indulgence of industrial and other human activities, it, in many areas, has become contaminated and unsafe for drinking purposes.^1,2^ Water pollution is now one of the emerging concerns, majorly caused by the consumption of essential medicinal drugs (e.g., ibuprofen) and discharge of textile industrial wastes (e.g., dyes).^2^ The toxicity and persistency of these contaminants in wastewater are particularly worrisome.^3,4^ Dyes are carcinogenic and very difficult to degrade, so their discharge into wastewater poses a great threat to human life and causes harmful effects on the environment. On the other hand, as one of the most commonly prescribed drugs in the world,^2^ the effect of ibuprofen on water contamination is also one of the worst. Moreover, similar to dyes, it is also difficult to remove from water via a conventional approach.^2^ There is an urgent need for the effective removal of these contaminants by an environmentally acceptable pathway involving no hazardous reactants or products to protect the environment and resources, notably water supplies.

Porous graphene (PG), a newly discovered promising substance for water treatment, was tested against trace concentrations of six new contaminants, namely, ciprofloxacin, diclofenac, atenolol, ibuprofen, gemfibrozil, and carbamazepine, and demonstrated a high removal efficiency (>99%) at its low dosage (100 mg/L)^3^ as well as good recyclability and effective regeneration (up to four cycles).^3^ Removal efficiency of the six pharmaceutical contaminants was effectively improved by using PG as a filter medium in an adsorption column filter tertiary unit.^5^ However, this PG was synthesized via the Hummers method, which involved noxious reactants, causing potential threat to the environment. Thus, the need for greener PG that is environmentally benign is illustrated in this study.

Apart from single-phase graphene, its composite counterparts performed well. For example, with a GNP/BN (graphene nanoplatelet/boron nitride) composite, a maximum adsorption capacity of 185 mg/g was achieved in the removal of ciprofloxacin,^6^ and in the case of using an Fe_3_O_4_/PG composite, a high adsorption capacity of 460 mg/g along with a rapid adsorption (within 5 min) was achieved against organic dye methyl violet.^7^

In the chemical synthesis of PG, graphite oxide (GO) is one of the key starting precursors.^8,9^ To make GO, a number of chemical processes are used, including the Staudenmaier–Hoffman–Hamdi method,^10^ modified Hummers method,^11^ and modified Hummers method and Tour’s method.^12^ However, each of these processes uses dangerous reactants, like sodium nitrate (NaNO_3_), and generates harmful byproducts, like perchlorate ions and dinitrogen tetra-oxide. The use of NaNO_3_ and perchloric acid (HClO_4_) in the modified Hummers method produces toxic gases like NO_2_ and N_2_O_4_ and explosive ClO_2_.^10,11^ Therefore, it is necessary to explore a more feasible and greener approach for GO synthesis.

Several methods using green reducing agents have been attempted to convert the GO synthesized by the Hummers method to graphene.^12^ In the cases of using hydrazine as a reductant, it could not be completely removed from the final graphene product.^11^ The emissions from it largely impact water toxicity and could give cancerous effects on humans.^13^ Gao et al. suggested that l-ascorbic acid (vitamin C) is an excellent alternative for hydrazine in the reduction of GO.^14,15^ Unlike hydrazine, l-ascorbic acid is environmentally friendly and does not cause any ill effects in human respiratory organs.^14,15^ Zengin Kurt et al.^16^ compared the effects of several reducing agents extracted from plants such as cloves, white mulberry, black cumin seed, blackthorn, dark grape, and rosehip on the synthesis of PG materials. However, all these relatively green techniques are only applicable to the synthesis of PG from a GO precursor. To our knowledge, there is no entirely green synthesis route from graphite to the intermediate GO and to the final PG. Recently, Peng et al. adopted a new green and low cost oxidizing agent, potassium ferrate (K_2_FeO_4_), to oxidize graphite, to form single-layered GO,^17^ and demonstrated the recyclability of sulfuric acid used and the economic viability of their process. Their GO was confirmed to be comparable to that formed using the Hummers method in terms of morphology and structure. In another study, the yield of GO from such a ferro-induced green synthesis was found to be higher (approx. 65%) than in the case of using the Hummers method (40%) without using a corrosive acid.^18^ Nevertheless, there is still a controversy over this ferro-induced GO synthesis, more specifically over the role and effectiveness of K_2_FeO_4_ as an oxidant. Sofer et al.^19^ claimed that K_2_FeO_4_ was unable to oxidize graphite and attributed the little oxidation (∼1.9% oxygen detected by XPS) of the graphite surface to some impurities such as KNO_3_ and KClO_3_ present in the original commercial K_2_FeO_4_. In another comparative study on the effects of different oxidizing agents,^20^ ferrate was found to be only capable of oxidizing graphite to low extents (10–15%).

If the above controversy could be clarified and, more importantly, if the high oxidizing capacity of K_2_FeO_4_ could be further confirmed or even improved, it would be able to make GO and graphene synthesis processes much greener and less expensive, given that it, as an oxidant, exhibits several advantages over potassium permanganate and other oxidants,^21^ including potentially high oxidation capacity, no risk of explosion, no hazardous byproducts, low cost, and ready availability. It is worth mentioning that K_2_FeO_4_ itself has already been widely used in water treatments.^22−29^

Our recent work has confirmed that the abovementioned controversy was actually caused by the rapid decomposition of K_2_FeO_4_ (within just several seconds) in air, making it unable to oxidize graphite completely. Therefore, it is necessary to inhibit or delay substantially the decomposition to achieve complete oxidation of graphite. To ensure that the decomposition/reaction of K_2_FeO_4_ proceeds in a more controllable and sustainable manner, a microencapsulation or coating strategy could be applied, as has been done previously with that used for direct wastewater treatment, in which case, several coating materials including ethyl cellulose, gelatine, chitosan, ethyl cellulose, and paraffin wax were used, and the coated K_2_FeO_4_ performed much better than its uncoated counterpart in the removal of methyl orange dye and dinitro butyl-phenol (DNBP).^21,22,28−30^

In this paper, we report for the first time on successful oxidation of graphite using encapsulated K_2_FeO_4_, which is an oxidant that exhibited a higher efficiency and greater degree of graphite oxidation. By using this near green method, high quality PG was further synthesized, which, acting as a promising adsorbent, demonstrated great potential for future water treatment applications that involved MeO dye and IBU emerging contaminants.

## 2 Materials and Methods

### 2.1 Raw Materials and Reagent Preparation

Powdered graphite (∼20 μm), H_2_SO_4_ (95.0–98%), paraffin wax, ethyl cellulose (48.0–49.8% (w/w)), and cyclohexane (anhydrous 95%) from Sigma-Aldrich were used to obtain MeO dye and analytical grade pharmaceutical IBU (Poole, Dorset, UK), and potassium ferrate was purchased from Skyrun Industrial Co. (Zhejiang, China)*.*

#### 2.1.1 Encapsulation of Potassium Ferrate (K_2_FeO_4_)

This was carried out by following the procedure reported in ref (25). Typically, 3 g of shell material (paraffin wax or ethyl cellulose) was added to a 50 mL cyclohexane solvent and magnetically stirred at 200 rpm at 60 °C until the former was completely dissolved. One gram of the core material, K_2_FeO_4_, was then added and further sonicated for 30 min. With the assistance of a peristaltic pump, the supernatant was removed, and the remaining material was dried at 80 °C in vacuum for 12 h to obtain the desired micro-encapsulated K_2_FeO_4_.

#### 2.1.2 Preparation of Graphene Oxide (GO)

**Figure 1:**
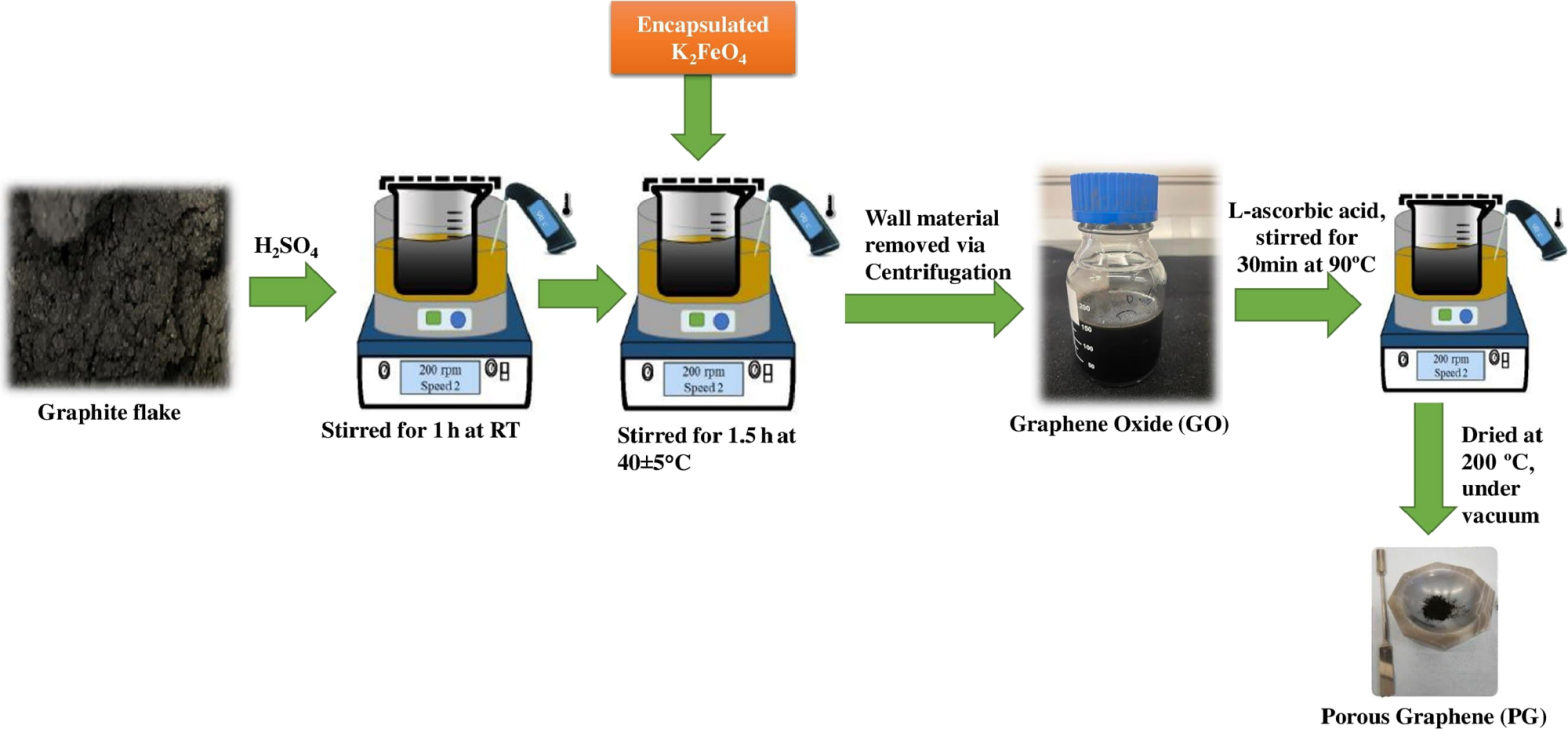
Schematic illustrating the near green synthesis process of novel greener PG.

Figure 1 illustrates the novel greener synthesis schematically, where GO was obtained when 0.5 g of graphite powder (∼20 μm) was intercalated for 1 h using concentrated sulfuric acid (95.0–98.0%) followed by addition of 4 g of the encapsulated oxidant (K_2_FeO_4_) prepared earlier. After 1.5 h of oxidation, the resultant suspension was centrifuged at 5300 rpm for 40 min. The remaining shell material was removed, and a brown colored supernatant (exfoliated GO) was collected and used directly (without the need of further sonication) for further PG preparation.

#### 2.1.3 Preparation of Porous Graphene (PG)

The GO prepared earlier was reduced with l-ascorbic acid (vitamin C) and subjected to heat treatment at 200 °C in vacuum to obtain PG.

IBU and MeO were combined with distilled water (DW) to create two stock solutions of contaminants, which were maintained in an airtight container and coated in aluminum foil to prevent unnecessary photodegradation. Batch tests used PG suspensions that had been sonicated for 30 min first.

### 2.2 Sample Characterization

After gold-coating and mounting on conductive carbon adhesive tapes, microstructures and morphologies of GO samples produced using bare K_2_FeO_4_, the modified Hummers method (replacing the permanganate oxidizing agent with K_2_FeO_4_), and the present near green synthesis with encapsulated K_2_FeO_4_ were examined in vacuum using a scanning electron microscope (TESCAN VEGA3 SEM) operated at an accelerating voltage 20 kV. Linked X-MAXN EDS was used to evaluate the content of oxygen-containing functional groups.

Absorbance spectra were recorded on a Fourier transform infrared spectrometer (FT-IR) (Bruker Optics Tensor-27) to analyze the oxygen-containing groups functionalities and arrangement of chemical bonds in GO. The spectra of GO resultant from the present near green route were compared with those of the GOs produced by the Hummers method and from a commercial source. The FT-IR was also used for the bare and encapsulated K_2_FeO_4_ in potassium bromide (KBr) pellet to identify the main peaks of ferrate(VI) and to evaluate the effectiveness of encapsulation. The samples were particularly opted to be mixed with KBr as it is transparent in the fingerprint IR region. Using 20 co-added scans, the absorbance spectra were acquired between 500 and 4000 cm^–1^ at a resolution of 4 cm^–1^. A total of 180 mg of KBr and 5 mg of each sample were thoroughly combined in a mortar before being manually pressed for 1 min at 5 MPa to create pellets. Then, each pellet was put in the sample holder for the FTIR analysis.

Further morphological examinations on the PG and GO samples were performed on a JEOL-2100 transmission electron microscope (TEM) running at a 200 kV accelerating voltage. After PG (Section S4) and GO were dispersed in ethanol and dropped onto the center of a holy carbon-copper grid, nanostructured images of the substances were obtained.

X-ray diffraction (XRD) patterns of GO and PG were recorded using an X-ray diffractometer from Bruker (D8 advanced) with Cu Kα radiation at 40 kV and 40 mA over a 2θ range of 10–80° with a scan rate of 2° (2θ). The GO UV–vis absorbance spectra were obtained (Section S1) at various dilution factors using a Jenway 6715 UV/Vis spectrophotometer. Thermal analysis was also carried out in Ar. Using a TA SDT Q600 TGA-DSC apparatus, thermal gravimetric analysis (TGA) profiles were collected at a ramping rate of 10 °C/min.

Raman scattering measurements were performed at room temperature (Section S2) on a Renishaw Qontor Raman spectrometer using a 50× objective, with a laser source of 532 nm excitation having 10% power, and 20 s integration time. Ten spectra were collected for each average (averaged spectra were background subtracted). A small amount of powder was spread out onto a glass slide, flattened, and then used to create the samples in this case.

The atomic force microscopy (AFM) images of PG (Section S3) were taken using a Bruker Innova AFM. A suspension of the sample (PG) in ethanol was prepared and then subjected to 30 min sonication. A few drops of the suspension were dropped onto a glass substrate before the AFM examination. The tapping mode was used to obtain the height profiles and images of the PG sample.

### 2.3 Batch Tests on Contaminant Removal

Batch tests were conducted for IBU and MeO under different conditions (such as different contact times and PG dosages). Each of them was done three times, and the average was calculated.

#### 2.3.1 Contact Time

IBU and MeO were the subjects of kinetic tests with beginning concentrations of 10.0 mg/L (standard solution) carried out across a range of time periods at room temperature (22 ± 3 °C) without the solutions’ pH levels being changed. A total of 20 mL of each of these contaminant solutions was poured into a 50 mL air-tight sealed bottle, and a given amount of PG (5 mg from a suspension of 5 g adsorbent/L) was added into these contaminant solutions. Each of the resultant solution was then magnetically stirred for a predefined time period (*t* = 5, 10, 15, 20, 40, 80, and 120 min). Finally, the samples collected at the defined time were filtered immediately through a 0.2 μm filter.

#### 2.3.2 Equilibrium Experiment

Batch tests were carried out to explore the effects of varying dosage of the adsorbent (0.5, 1, 1.5, and 2 mL dosages of PG injected from a 5 g/L suspension) injected into a certain contaminant’s concentration (10 mg/L IBU and MeO). A total of 20 mL of each of the contaminants with different PG dosages was placed into a 50 mL tube on a shaker for 24 h, and the samples were collected via vacuum filtration.

## 3 Results and Discussion

### 3.1 Comparison of Encapsulated and Bare K_2_FeO_4_

**Figure 2:**
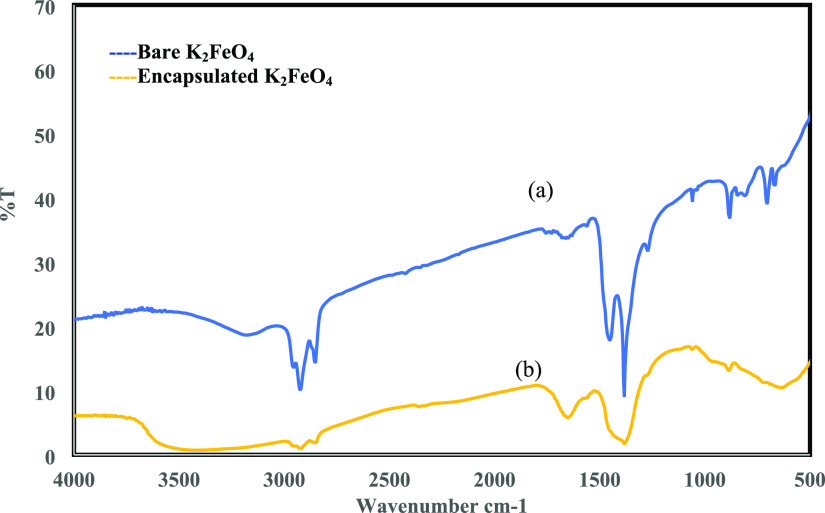
FTIR spectra of (a) bare K_2_FeO_4_ and (b) encapsulated K_2_FeO_4_.

**Figure 3:**
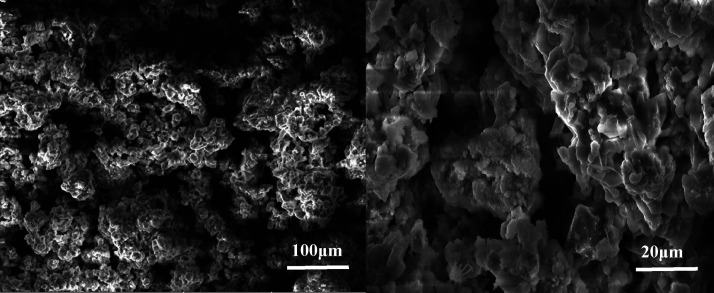
SEM images of bare K_2_FeO_4_.

**Figure 4:**
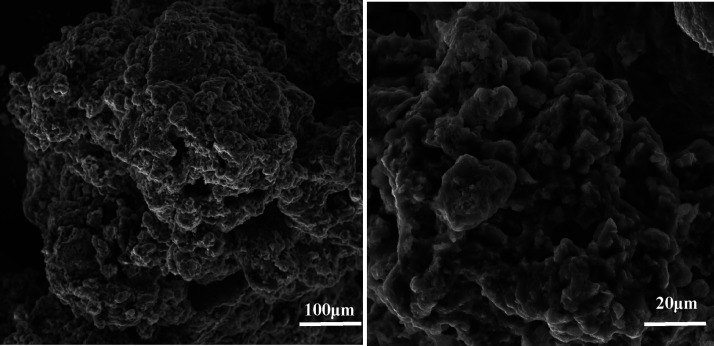
SEM images of encapsulated K_2_FeO_4_.

Figure 2 presents together the transmittance FT-IR spectra of bare and encapsulated K_2_FeO_4_. In the case of the former (Figure 2a), a primary peak appeared at 808 cm^–1^ along with a shoulder peak at 780 cm^–1^, which was the characteristic peak of FeO_4_.^2−24^ In the case of the latter (Figure 2b), the spectrum showed the characteristics peaks of the shell materials. The characteristic peak at 1100 cm^–1^ arose from ethyl cellulose, and those centered around 2916, 1471, and 720 cm^–1^ belong to the stretching and bending vibrations of paraffin wax.^24^ Also, the spectrum showed the peaks of K_2_FeO_4_, indicating that the encapsulation was intact. The SEM images of bare K_2_FeO_4_ and encapsulated K_2_FeO_4_ are shown in Figures 3 and 4, respectively. In the case of the latter, smooth surfaced particles containing pores are seen, assisting in the slow release of the oxidant when added to graphite.

### 3.2 Effect of Encapsulation of K_2_FeO_4_ on the GO Formation

K_2_FeO_4_ is a dark purple powder and does not decompose in dry air or a moisture-free environment. In an acidic or a neutral solution, it reacts with water, releasing oxygen gas and forming ferric complexes,^31^ as expressed by eq 1:^31^
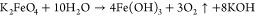
1

When concentrated sulfuric acid is present, however, K_2_FeO_4_ decomposes extremely rapidly, typically within several seconds. For this reason, when bare K_2_FeO_4_ is used as an oxidant, it will not be able to completely oxidize graphite, so only partially oxidized graphite oxide (GIO) will be formed, as indicated by eq 2(17)
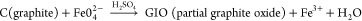
2

**Figure 5:**
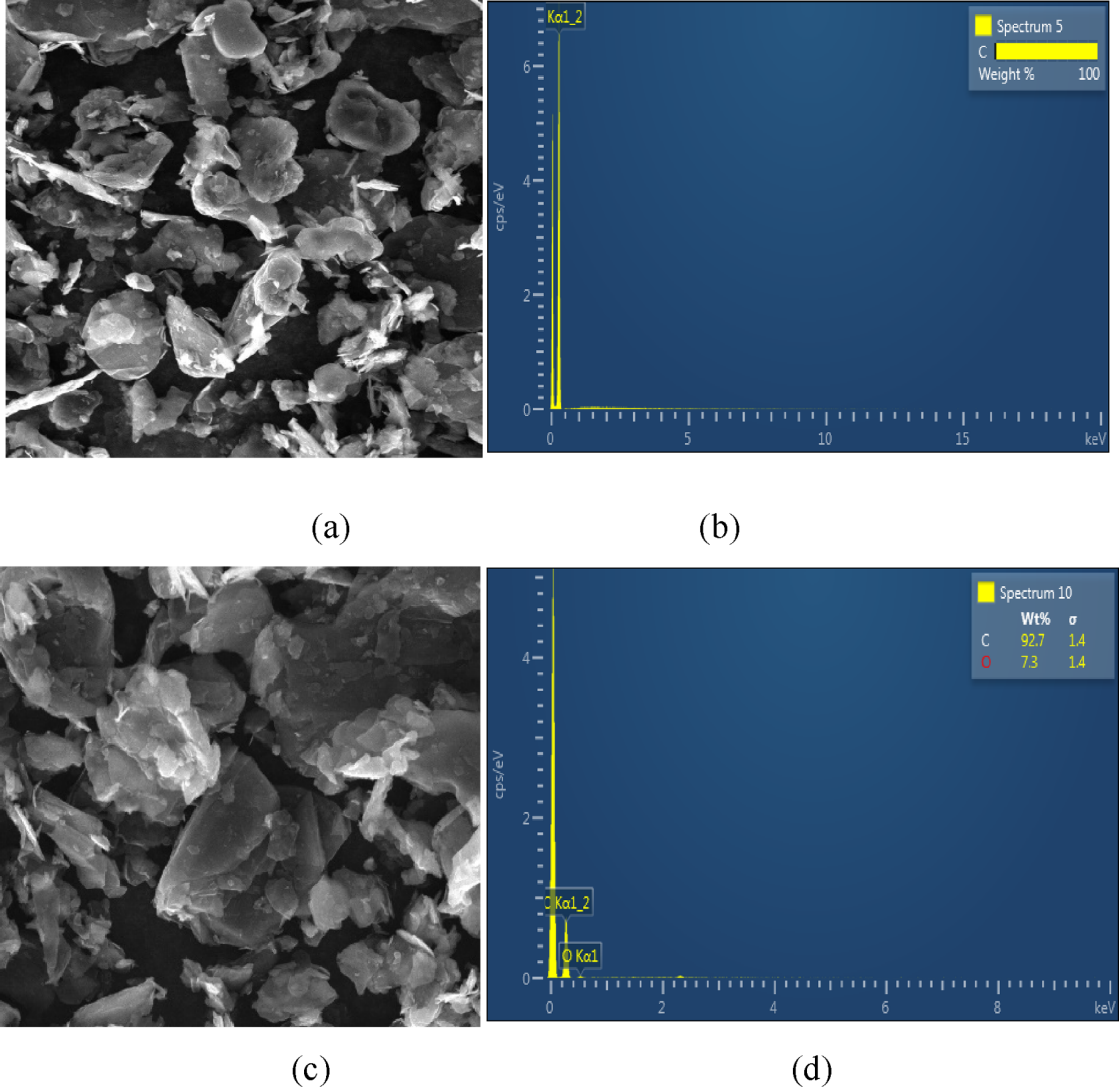
SEM-EDS images of (a, b) pristine graphite and (c, d) partially oxidized graphite oxide, obtained from oxidation via bare K_2_FeO_4_.

**Figure 6:**
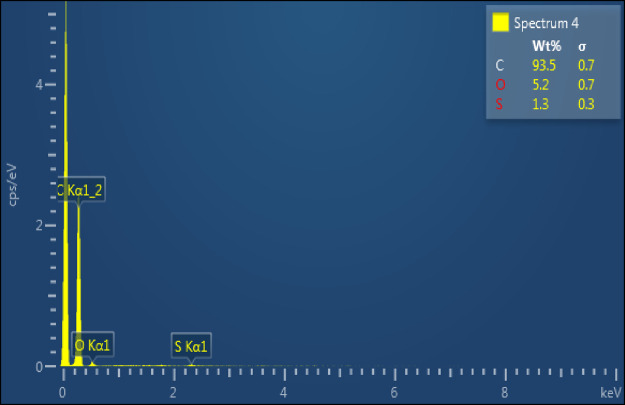
EDS spectrum of partially oxidized graphite oxide, resulting from replacing the KMNO_4_ used by the modified Hummers method with bare K_2_FeO_4_.

This explains well the results reported in ref (19) and was further verified by the SEM observations and EDS results from the pristine graphite powder (Figure 5a,b) and the graphite sample after being oxidized by bare K_2_FeO_4_ at a lower temperature of *T* = 35 ± 5 °C (Figure 5c,d). As expected, only the C element was detected by EDS in the case of the former, indicating the high purity of the graphite precursor. In the case of the latter, the phase morphology and size, to a large extent, were still retained, but EDS identified the coexistence of 92.7% carbon and 7.3% oxygen in the sample, indicating that the original graphite was only slightly oxidized to GO by K_2_FeO_4_. These results confirmed again that uncoated K_2_FeO_4_ only had a limited oxidizing capacity in graphite oxidation; in other words, the complete oxidation of graphite would not be achieved if bare K_2_FeO_4_ was used as an oxidant, as previously considered by Sofer et al.^19^ On the other hand, in the case of GO preparation via the modified Hummers method using bare K_2_FeO_4_ to replace the conventionally used KMNO_4_ oxidant, almost identical results to those of Sofer et al.^19^ were obtained. As revealed by EDS (Figure 6), in this case, the carbon content remained as high as 93.5% and the oxygen content was as low as 5.2% (the minor sulfur (Figure 6) detected was due to the contamination from the concentrated sulfuric acid used). This additionally verified the poor oxidizing ability of bare K_2_FeO_4_ to oxidize graphite.

**Figure 7:**
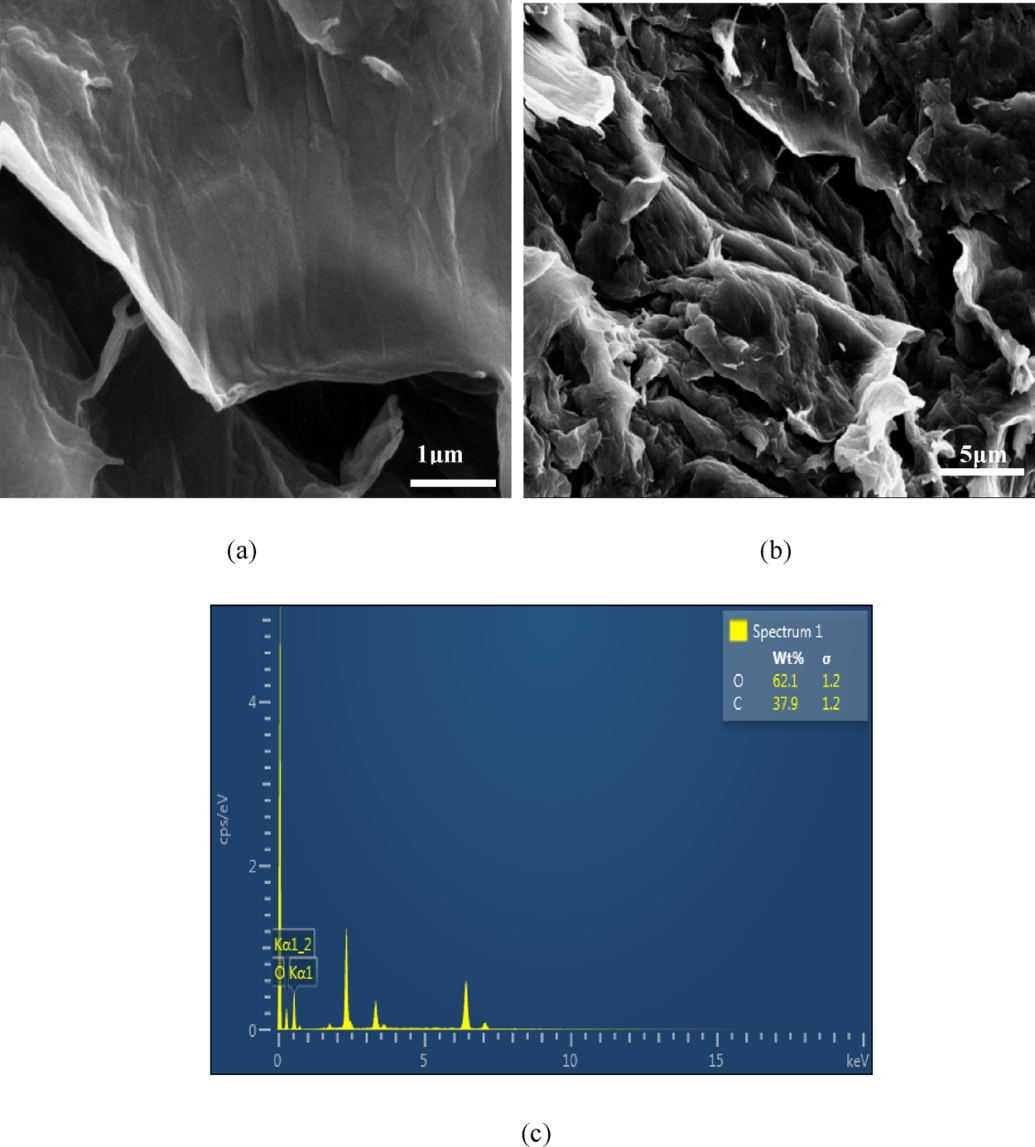
(a, b) SEM images and (c) EDS spectra of GO resulting from oxidation treatment at a relatively high temperature of 40 °C by using paraffin wax- and ethyl cellulose-coated K_2_FeO_4_.

Presented in Figure7 are the SEM image and EDS of a GO sample resulting from the oxidation treatment at a relatively high temperature (*T* = 40 ± 5 °C) using encapsulated K_2_FeO_4_ as an oxidant, revealing clearly the formation of GO sheets with lateral sizes up to 5 μm (Figure 7a,b) and the presence of as high as 62.1% oxygen along with only about 37.9% carbon, suggesting much higher extents in the graphite oxidation and in the conversion from graphite to GO. The average C/O ratio in the GO sample was calculated to be 1.02 based on the carbon and oxygen contents determined by EDS. This value was reasonably close to the theoretical one (1.5) for pure GO, indicating that the encapsulated K_2_FeO_4_ was a much more effective oxidant for GO preparation than its uncoated counterpart.

**Figure 8:**
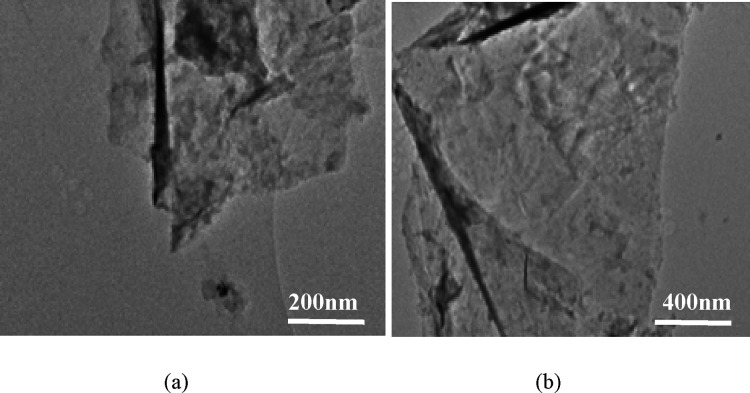
(a) Low and (b) high magnification TEM images of GO prepared by the near green synthesis using coated K_2_FeO_4_.

Figure 8 further gives the TEM images of the GO sample prepared using encapsulated K_2_FeO_4_, confirming the formation of GO nanosheets with well-defined bilayers at the edges and some “intrinsic” wrinkles.

**Figure 9:**
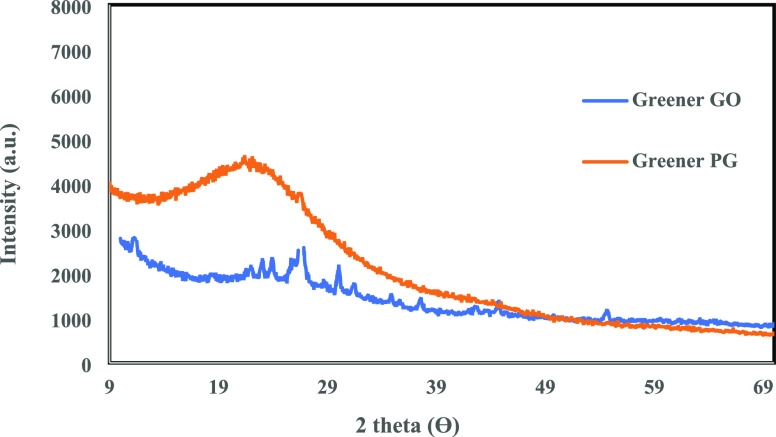
XRD spectrum of the GO produced by using the near green synthesis using encapsulated K_2_FeO_4_ as an oxidant and XRD spectrum of the PG resulting from reduction of the GO prepared via the near green process by vitamin C.

Given in Figure 9 is the XRD pattern of the GO sample resulting from the near green synthesis at 40 ± 5 °C, using coated K_2_FeO_4_ as an oxidant, showing a diffraction peak at 11.5° (2θ), which is corresponding to a *d*-spacing of 0.77 nm of GO.^32^ However, another peak appeared at 24° (2θ), which arose from the residual shell materials, paraffin wax and/or ethyl cellulose. The former has a sharp characteristic XRD peak at 24.85° (2θ),^33^ and the latter has a broad one within 20–26° (2θ).^34^ Thus, the formation of a well-exfoliated GO structure via the newly developed near green synthesis route was confirmed additionally.

**Figure 10:**
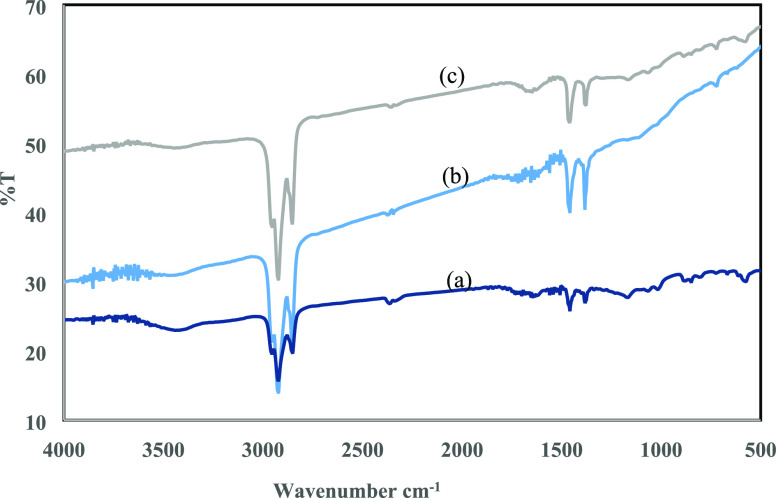
FT-IR spectra of GO samples resulting from (a) the near green route, (b) the modified Hummers route, and (c) the commercial source.

As demonstrated in Figure 10, GO prepared from the present near green route (Figure 10a) exhibited a similar spectrum to its counterpart prepared from the modified Hummers route (Figure 10b) or from a commercial source (Figure 10c). Functional groups in GO were discovered as C–O (1240 cm^–1^), C-O-C s (1050 cm^–1^), O–H (3412 cm^–1^),^35−38^ C=O (1726 cm^–1^), stretching vibrations, and C=C from sp2 bonds within the range of 3500 to 500 cm^–1^ (1624 cm^–1^). Additionally, the symmetric and asymmetric stretching vibrations of the C–H bond were responsible for the bands at 2855 and 2920 cm^–1^, respectively. The FT-IR spectrum of GO prepared from the near green synthesis route identified the same peaks as those of the GO from the commercial source or from the modified Hummers synthesis process.

The UV–visible spectrum of as-prepared GO is shown in Figure S1, in which a peak appeared at 250–260 nm. Such a peak shift from 230 nm was due to the coexistence of GO with some impurity phases,^39,40^ residual wall material, and sulfuric acid. A similar shift of the GO peak from 230 to 270 nm was also observed previously,^39^ which was considered to arise from another impurity phase.

**Figure 11:**
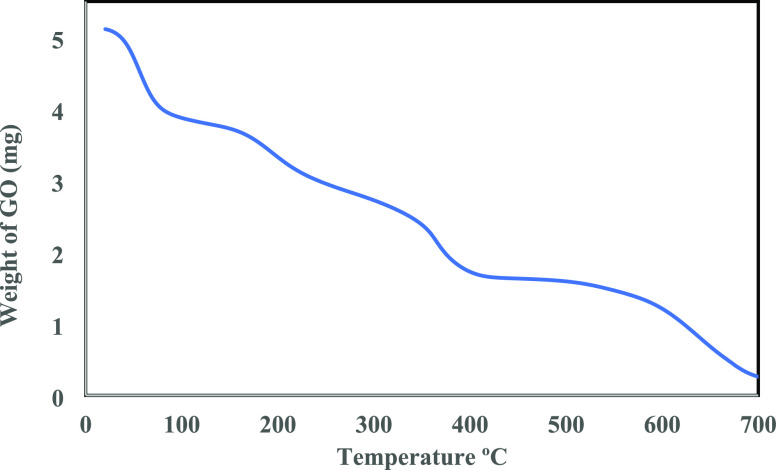
TGA curve of the GO prepared via the near green route using encapsulated K_2_FeO_4_ as an oxidant.

Figure 11 further provides the TGA profile of as-prepared GO. The four peaks in the curve correspond to the following thermal events: water evaporation contributed the weight loss below 100 °C; the significant weight loss at 100–360 °C arose from the loss of oxygen functional groups in GO; the weight loss between 360 and 700 °C was associated with the oxidative pyrolysis of a carbon network; and the last peak was due to the removal of highly stable functional groups (indicating higher oxidation of the graphite material).

### 3.3 Formation of Porous Graphene from As-prepared GO

Figure 9 shows the XRD pattern of the PG sample prepared using the GO synthesized via the near green route and vitamin C as a precursor and a reductant, respectively. The absence of the peak at 2θ ∼ 11.5° and the appearance of a broader peak around 2θ ∼ 24° corresponding to the (002) plane suggest the successful reduction of GO to graphene by vitamin C.

**Figure 12:**
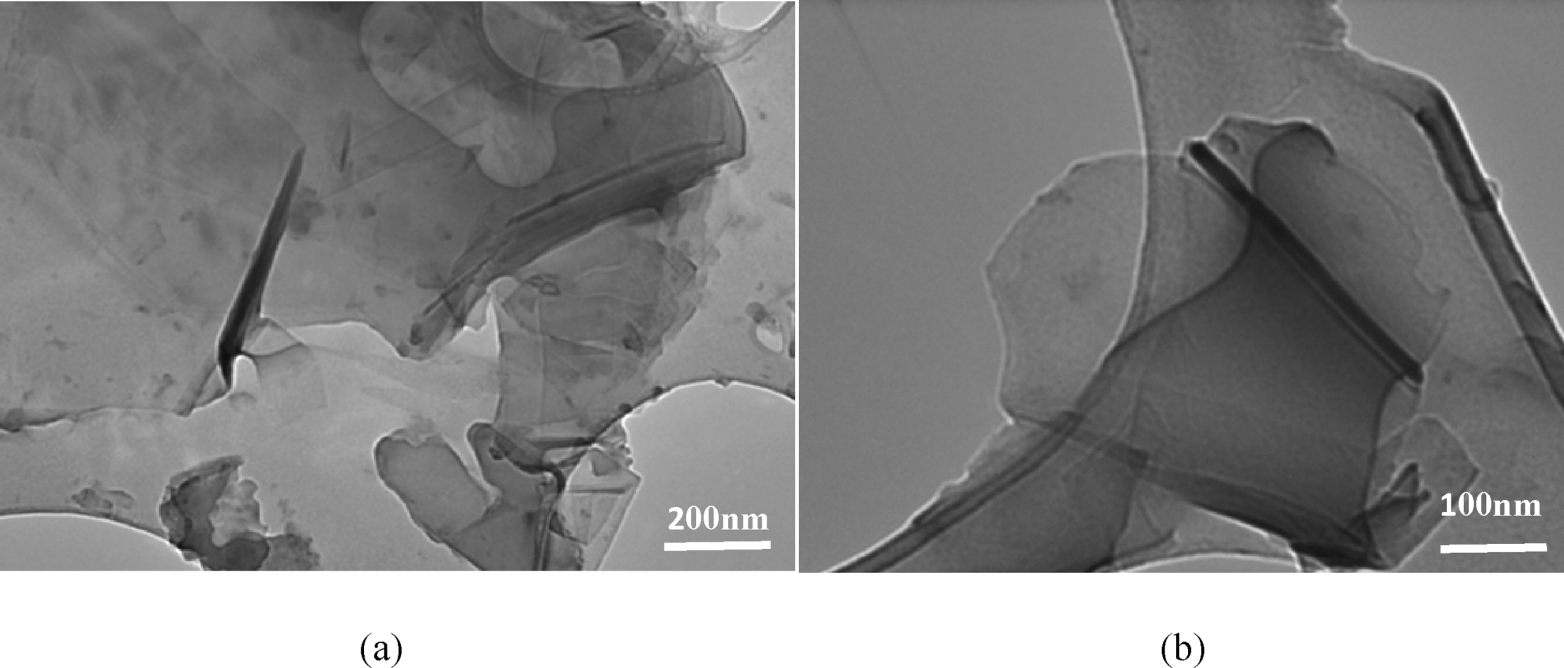
(a) Low and (b) high magnification TEM images of porous graphene prepared via the near green route.

The morphology of the PG sample was also examined with TEM (Figure 12), revealing a multilayered (bilayered) graphene sheet structure. This was further confirmed by AFM (Figure S3) and Raman spectra (Figure S2). The morphology of the PG was also examined by SEM (Figure S5a–c), revealing the presence of pores on the surface of a well-exfoliated multilayer graphene material that was further proven to contain more than 90% carbon by EDS (Figure S5d). Structural changes corresponding to the subsequent reduction of GO to PG were studied based on the Raman spectra (Figure S2). The two peaks corresponding to the G-band and D-band were identified, with the ratio of *I*_D_/*I*_G_ = 0.344. According to the literature, for multilayered graphene, the ratio of *I*_2D_/*I*_G_ should be <0.4.^41−43^ Here, the *I*_2D_/*I*_G_ ratio was found to be 0.108, showing that the reduction of GO by vitamin C resulted in the formation of multi- or bilayered graphene. Figure S3, from AFM analysis, shows that the thickness of the synthesized graphene was ∼1 nm, further verifying the formation of multilayered/bilayered graphene. Such a stacking might be due to the absence of any stabilizing agent for graphene.

### 3.4 Adsorption Tests

#### 3.4.1 Effect of Contact Time on Contaminant Adsorption

**Figure 13:**
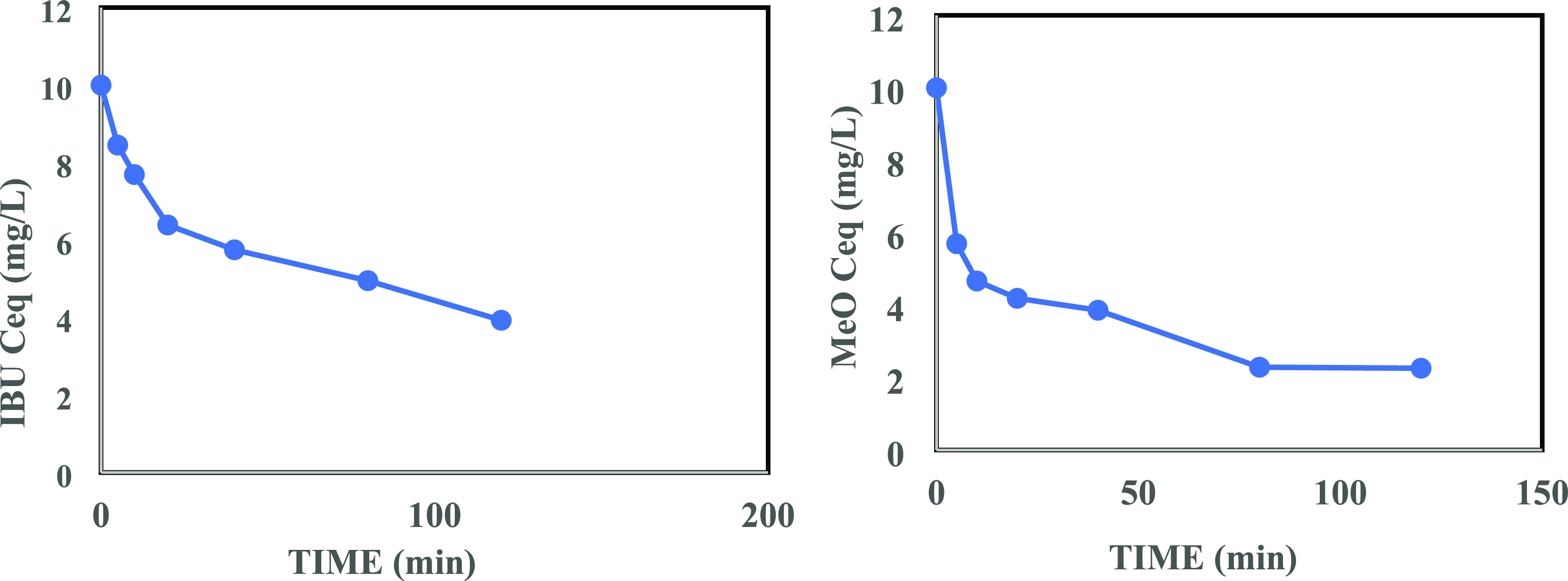
Effect of contact time on adsorption of IBU (10.0 mg/L) onto the as-prepared PG (1.0 mL dose from 5 g/L suspension).

**Figure 14:**
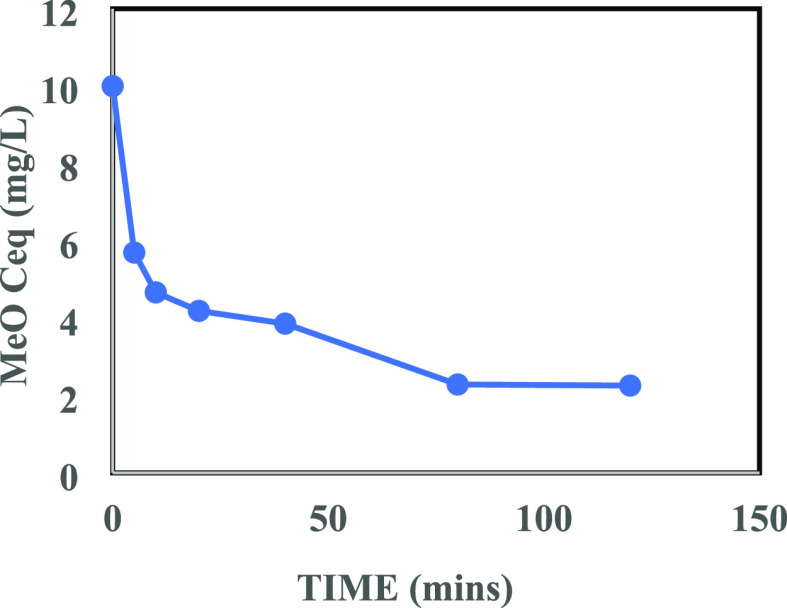
Effect of contact time on adsorption of MeO (10.0 mg/L) onto the as-prepared PG (1.0 mL dose from 5 g/L suspension).

Figures 13 and 14 show, respectively, the concentrations of IBU and MeO (from an initial concentration of 10.0 mg/L conc.) as a function of contact time. The initial 60 and 40 min periods were characterized by fast sorption kinetics, i.e., a high rate of adsorption, for contaminants IBU and MeO, respectively, after which the adsorption rate plummeted with a further increase in the contact time. These results showed an excellent adsorption performance of the as-prepared PG for these organic pollutants, which could be attributed to the pores on its surface (Figure S4b).

#### 3.4.2 Effect of Adsorbent Dosages on Contaminant Removal

**Figure 15:**
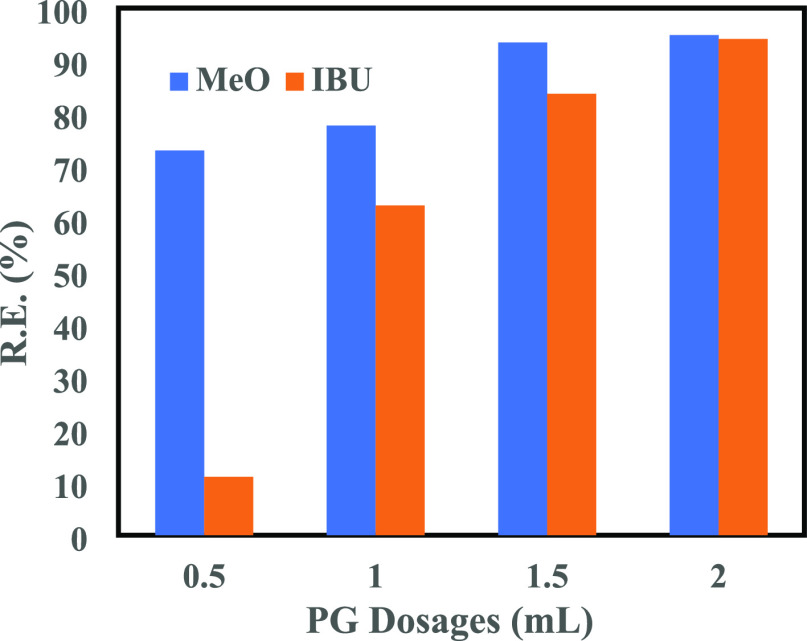
Removal efficiencies for IBU and MeO as a function of PG dosage.

To evaluate the removal capacity of the as-prepared PG, batch adsorption tests were carried out using four different dosages of PG (0.5, 1, 1.5, and 2 mL of adsorbent injected from a 5 g/L suspension). The test time in each case was 24 h to ensure that the equilibrium was reached. Figure 15 illustrates the effects of PG dosage on the removal of IBU and MeO (10 mg/L conc.) in terms of their removal efficiency (R.E.). It was evident that with increasing the adsorbent dosage, the removal efficiency increased. The 2 mL PG dosage showed the highest removal efficiency (>90%), which was resulting from the hydrophobe–hydrophobe attraction between the PG and the contaminants. At this dosage, removal efficiencies of 94.4 and ∼95% were achieved for IBU and MeO, respectively, suggesting that the PG prepared via the present near green route could be an excellent candidate adsorbent for future water treatment applications.

**Figure 16:**
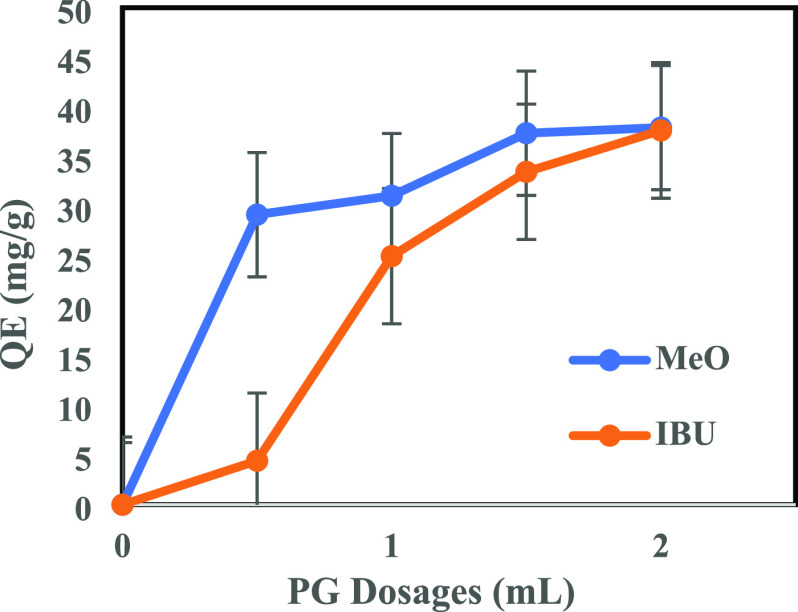
Adsorption curves in the cases of IBU and MeO removal.

The removal efficiencies (from Figure 15) of IBU and MeO were further explained by the corresponding maximum adsorption capacities (QE) at 2 mL PG dosages (Figure 16), which reached 37.8 and 38 mg/g, respectively.

## 4 Conclusions

Graphene oxide was successfully synthesized using a novel near green technique, as verified by detailed microstructural and compositional characterizations. This greener GO synthesis technique used a K_2_FeO_4_ oxidant encapsulated by a shell material to enable its slow release. As-prepared green GO was further used as a precursor to form PG via chemical reduction with vitamin C and subsequent thermal reduction at 200 °C. Raman spectroscopy showed the presence of two peak bands “D” and “G”, with an *I*_2D_/*I*_G_ ratio of 0.108, demonstrating the formation of multilayered/bilayered PG, which was additionally verified by AFM.

Compared to the conventional Hummers method, the greener synthesis route developed in this work exhibited several advantages such as shorter oxidation time and much less consumption of oxidizing agent. To illustrate its potential for future applications, as an example, the PG synthesized via the present greener route was tested for the removal of MeO and IBU contaminants. In both cases, the removal efficiency reached >90%, suggesting that the as-prepared PG could be potentially used as a strong candidate adsorbent material for future wastewater treatments.

## Supporting Information Available

UV–vis result, Raman result, AFM result, PG-SEM result, and XRD of GO (PDF)
