# Supplementary material for: Near Green Synthesis of Porous Graphene from Graphite Using an Encapsulated Ferrate(VI) Oxidant
Source: ACS Omega. 2023 Aug 3;8(32):29674–84. doi: 10.1021/acsomega.3c03812 (PMC10433472; doi:10.1021/acsomega.3c03812)
Supplement: Supplementary file 1 — ao3c03812_si_001.pdf [file ao3c03812_si_001.pdf]

SUPPORTING INFORMATION

Near Green Synthesis of Porous Graphene from Graphite Using an Encapsulated Ferrate (VI) Oxidant

Bhavya Joshi [a*#], Ahmed M.E. Khalil[a,b#], Tanveer A. Tabish[c], Fayyaz A. Memon[a], Hong Chang[a], Shaowei Zhang[a*]

[a] Faculty of Environment, Science and Economy, University of Exeter, UK EX4 4QF

[b] Department of Chemical Engineering, Faculty of Engineering, Cairo University, Giza 12613, Egypt

[c] Division of Cardiovascular Medicine, Radcliffe Department of Medicine, University of Oxford, Oxford, OX3 7BN, United Kingdom

# Authors contributed equally

* Corresponding authors' e-mail addresses: bj300@exeter.ac.uk, s.zhang@exeter.ac.uk

S1. UV-vis Result

UV-visible spectra of as prepared graphene oxide is shown in Figure S1**.** It shows a presence of a peak around 250-260nm.

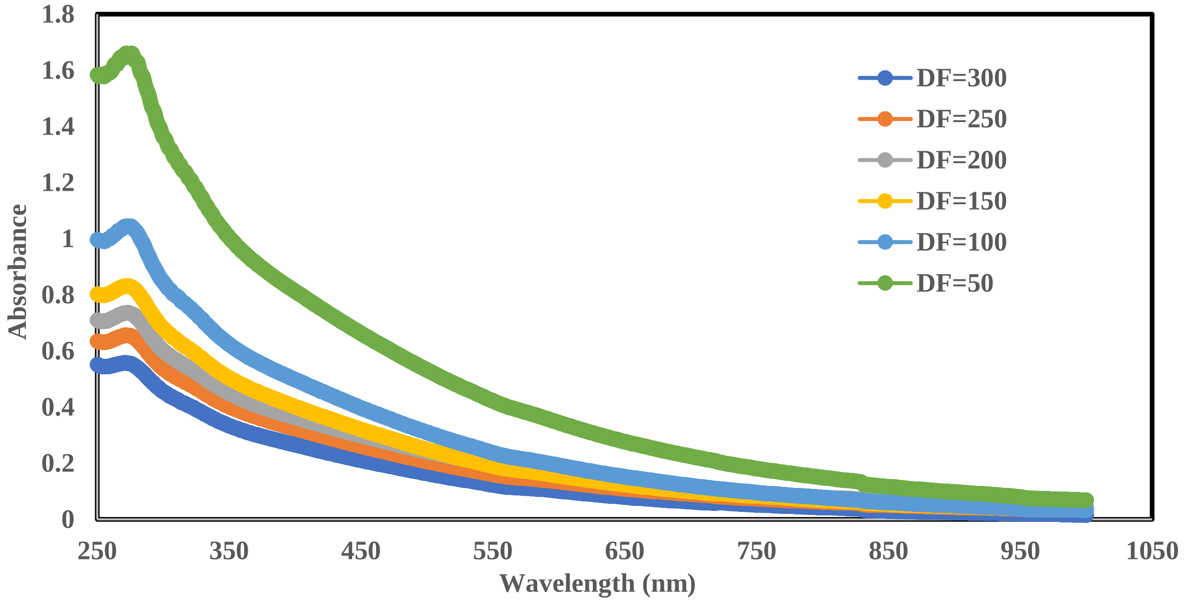

Figure S1: Absorbance *Vs* Wavelength plot for Green-Graphene Oxide for different dilution factor. (UV-vis peak in range 250-260 nm).

S2. RAMAN RESULT

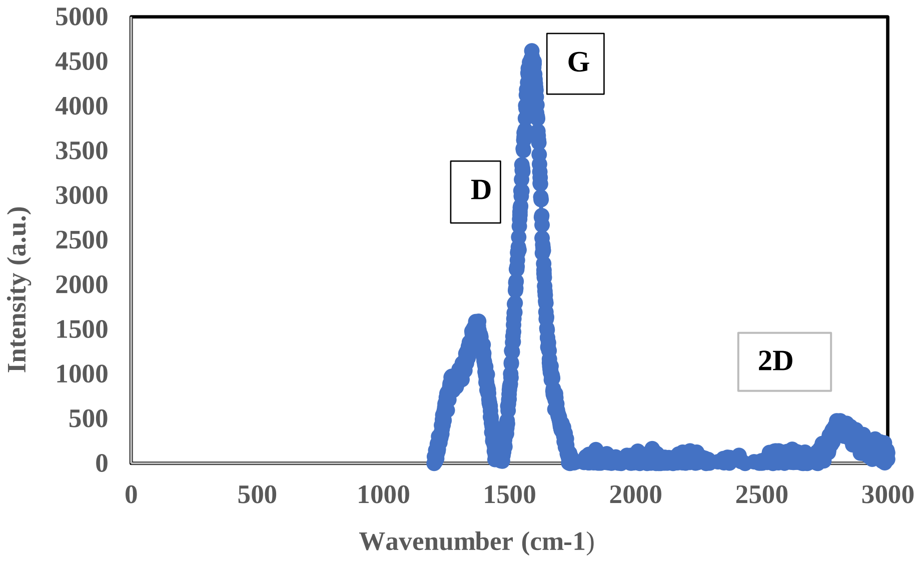

Figure S2: Raman Spectra of Prepared green graphene with I_{2D}/I_g ratio=0.108.

S3. AFM RESULT

Number of graphene layers calculated ~2 , indicating bi-layer graphene ,by the following formulae [1]:
N= ($T_{measured}$-0.4)/0.335

where, N=number of graphene layers

$T_{measured}$= measured thickness via AFM instrument

0.4= account for increases in measured thickness related to substrate – graphene and graphene–tip interactions
0.335= inter-plane spacing of graphite

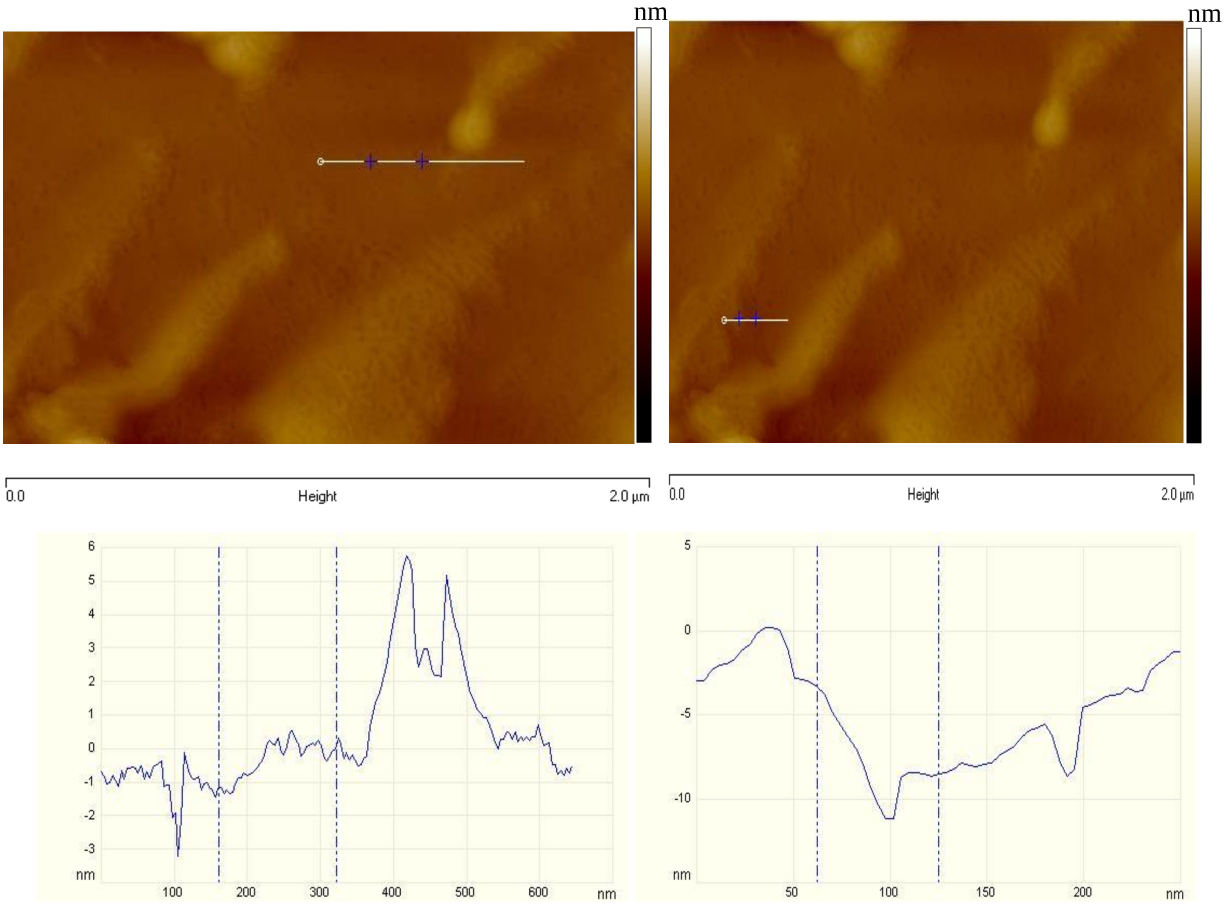

Figure S3: AFM image of as-prepared green graphene with its respective height profiles.

S4. PG-SEM RESULT

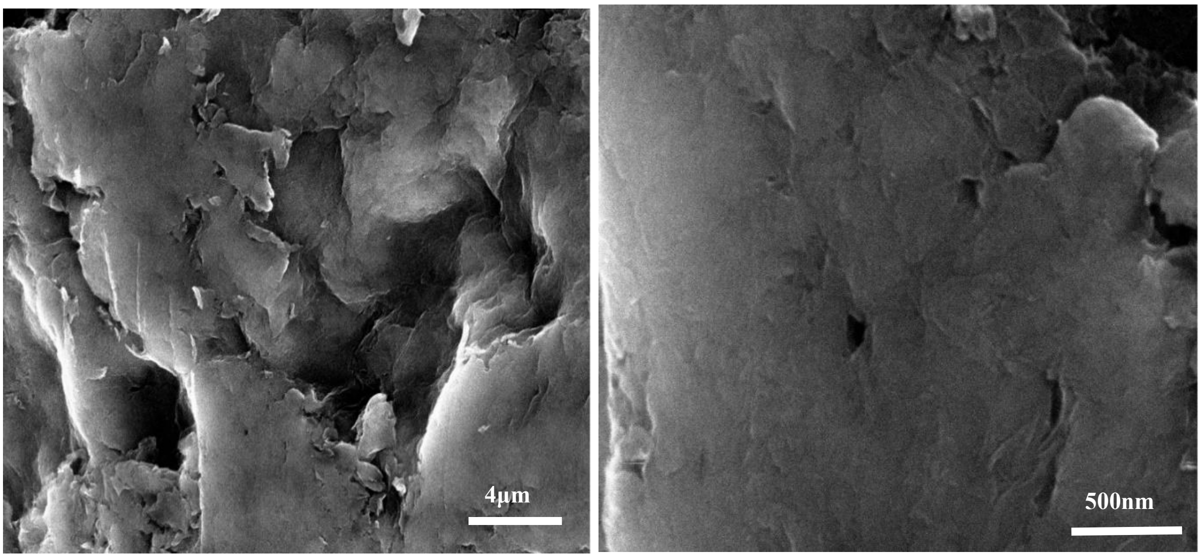

(a) (b)

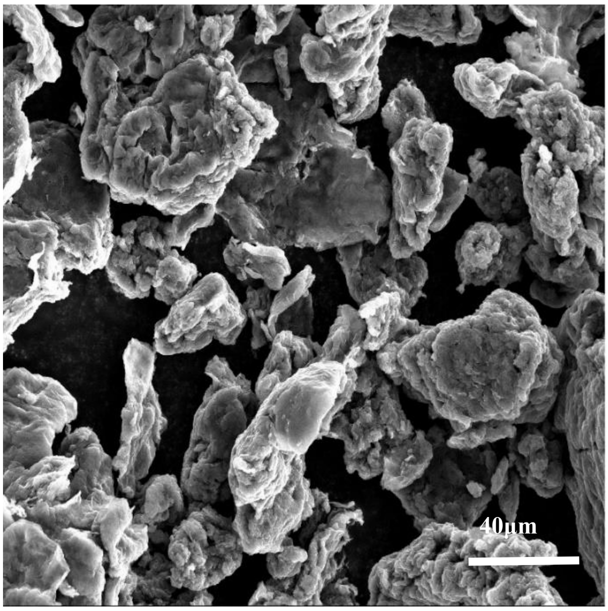

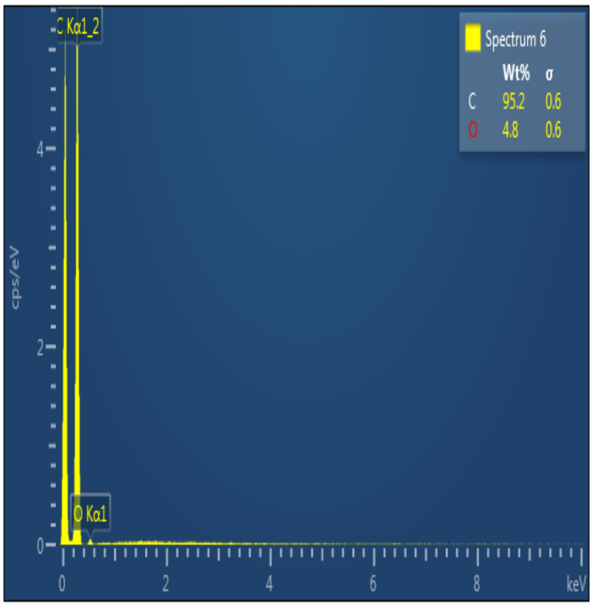

(c) (d)

Figure S4: a-c) SEM images of as-prepared graphene material; d) EDS analysis of as-prepared graphene material.

S5. XRD of GO: Obtained from bare K_2FeO_4.

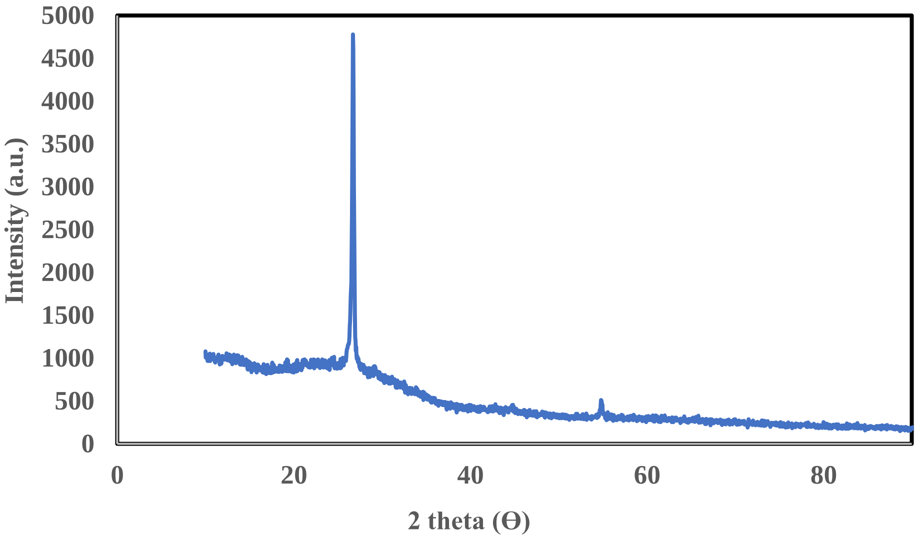

Figure S5: XRD spectrum of the GO produced bare K_2FeO_4.

REFERENCES:

(1) Shearer, C. J.; Slattery, A. D.; Stapleton, A. J.; Shapter, J. G.; Gibson, C. T. Accurate Thickness Measurement of Graphene. *Nanotechnology* **2016**, *27* (12). https://doi.org/10.1088/0957-4484/27/12/125704.
